# The power of microbes: the key role of gut microbiota in the initiation and progression of colorectal cancer

**DOI:** 10.3389/fonc.2025.1563886

**Published:** 2025-04-14

**Authors:** Bo Han, Yongfeng Zhang, Xue Feng, Jun Yang, Baolin Wang, Jiang Fang, Zhigang Wang, Jun Zhu, Ge Niu, Youxiang Guo

**Affiliations:** ^1^ Department of General Surgery, 63650 Military Hospital, Urumqi, China; ^2^ Department of Cardiology, 63650 Military Hospital, Urumqi, China

**Keywords:** gut microbiota, colorectal cancer, chronic inflammation, metabolites, immune dysfunction, probiotics, natural plant extracts

## Abstract

Colorectal cancer (CRC) is ranked as the third most prevalent malignancy and is a leading cause of cancer-related mortality globally, significantly affecting the health and longevity of middle-aged individuals and the elderly. The primary risk factors for CRC are mainly due to unhealthy dietary habits and lifestyle choices, and they have been shown to profoundly influence the composition of the gut microbiota. Given that dietary patterns are critical determinants of gut microbial diversity, a compelling association exists between gut microbiota and the pathogenesis of CRC. Recent research has increasingly focused on the intricate interplay between gut microbiota and CRC, exploring its role in disease initiation, progression, and the modulation of host immune responses. Investigations have demonstrated that certain specific microbial communities can promote inflammation, disrupt metabolic pathways, and produce carcinogenic compounds, thereby contributing to the development of CRC. Conversely, a diverse and balanced gut microbiome may confer protective effects against cancer through mechanisms such as the production of short-chain fatty acids and the enhancement of intestinal barrier integrity. This article provides a comprehensive overview of the characteristics of the gut microbial community and its complex relationship with CRC. It highlights potential mechanisms through which gut microbiota may influence CRC pathogenesis, including chronic inflammation, toxins, metabolites, epigenetic dysregulation, and immune regulatory dysfunction. Additionally, this review summarizes innovative strategies for CRC prevention and treatment, emphasizing the therapeutic potential of probiotics and natural plant extracts. By elucidating these connections, this work aims to enhance the understanding of the gut microbiome’s role in CRC.

## 1 Introduction

Colorectal cancer (CRC), originating from the mucosal cells of the colon and rectum, is one of the most prevalent malignancies of the digestive system, predominantly affecting individuals aged 50 and older (1, 2). Recent epidemiological data indicate a concerning increase in both the morbidity and mortality rates associated with CRC (3). Globally, CRC is ranked as the third most common cancer, with over 1.93 million new cases and approximately 935,000 deaths reported in 2020, accounting for about 10% of all cancer incidences and 9.4% of cancer-related deaths, making it the third leading cause of death in both male and female groups (4–6). The number of new CRC cases worldwide may rise to 2.5 million by 2035, surpassing liver and stomach cancers (7). CRC typically exhibits a slow growth pattern, progressing gradually from small, inconspicuous adenomas to malignant tumors over several years, with potential for metastasis to the whole body (8). In its early stages, CRC is often asymptomatic; however, advanced cases may present with symptoms such as abdominal pain, changes in bowel habits, hematochezia, and intestinal obstruction (9, 10).

The etiology of CRC involves both genetic and environmental factors. While genetic factors, such as germline mutations, account for a small proportion of cases, most instances are sporadic, suggesting a complex interaction between genetics and environmental influences in CRC development (11, 12). Key risk factors include unhealthy lifestyle choices and dietary habits, such as smoking, excessive alcohol consumption, physical inactivity, obesity, high intake of red and processed meats, and low consumption of fiber-rich foods (13). In particular, dietary habits are significantly associated with the incidence of CRC (14). Notably, dietary patterns considerably influence the composition of gut microbiota (15, 16), which has been increasingly recognized for its roles in the pathogenesis and progression of CRC (17–20).

Trillions of microorganisms in the human gut form a complex microecosystem that regulates interactions between the host and the environment. This ecosystem plays a crucial role in digestion, metabolism, nutrient absorption, immune regulation, and pathogen defense, significantly impacting overall health and disease states (21–23). The normal gut microbiota maintains the homeostasis of the intestinal environment, thereby protecting the structure and function of mucosal epithelial cells (24). Conversely, dysbiosis in gut microbiota can lead to a reduction in beneficial microorganisms and an increase in pathogenic microbes, subsequently altering the intestinal microenvironment. Consequently, this alteration may activate inflammatory responses, produce toxic metabolites, induce gene mutations, and damage the intestinal epithelial barrier (25, 26). Numerous studies have demonstrated that both structural changes and functional disorders of gut microbiota are closely related to the development of CRC (27–30).

Compared with healthy individuals, patients with CRC exhibit dysbiosis characterized by a decrease in the abundance of bacteria with potential protective effects and an increase in pathogenic bacteria (18, 24, 31). In the context of gut microbiota dysbiosis, pathogenic bacteria replace non-harmful symbiotic bacteria, triggering host inflammation, promoting cellular proliferation, and inducing oncogenic signals along with metabolic byproducts, which influence the development of CRC (24, 32). In addition to bacteria, fungal pathogens are also reported to trigger the process of colorectal carcinogenesis (33).

Moreover, the gut microbiota has a dual role in CRC. It may promote both the initiation and progression of the disease through various mechanisms. At the same time, it can also exert inhibitory effects on tumor activity (34). For instance, enterotoxigenic *Bacteroides fragilis* can induce inflammatory diarrhea and promote CRC progression through the secretion of *Bacteroides fragilis* toxin (BFT) (35). In contrast, *Lactobacillus* can reduce the risk of CRC by secreting short-chain fatty acids (SCFAs) and other metabolites (36). *Candida albicans* can trigger the upregulation of glycolytic levels in macrophages, leading to the secretion of IL-7 and ultimately promoting the production of IL-22 by innate lymphoid cells type 3 (ILC3). This cascade effect enhances the proliferation of intestinal epithelial cells and contributes to the development of colitis-associated colon cancer (CAC) (37, 38). However, certain commensal fungal species possess anti-inflammatory properties and the ability to modulate the immune response (39). For example, *Saccharomyces cerevisiae* can mitigate AOM/DSS-induced CRC by modulating the intestinal microbiota and lowering the levels of pro-inflammatory mediators (40). Additionally, improving gut microbiota composition can increase the proportion of beneficial microorganisms, thereby effectively retarding the progression of CRC (41).

This article aims to summarize the latest advancements in this field. It will elucidate the relationship between gut microbiota and CRC, as well as the potential mechanisms involved. The goal is to provide valuable targets for the prevention and treatment of CRC in clinical practice.

## 2 Characteristics of the human gut microbiota

The human gut is home to a vast array of microorganisms, including bacteria, archaea, viruses, fungi, and protists, characterized by their dense distribution and active metabolism. Collectively, these organisms form a complex and diverse microecosystem that plays a crucial role in various physiological and pathological processes (42, 43). Bacteria constitute the most abundant group of microbes in the intestines, numbering approximately 10^13, which is estimated to be ten times the total number of host cells. The total genomic content of bacteria exceeds that of the host by more than 100-fold, which has led to its classification as the second-largest gene pool in the human body (44, 45). In healthy adults, the dominant intestinal flora include *Firmicutes* and *Bacteroidetes*, which together account for 90% of the gut microbial community. Most of these dominant groups of bacteria are anaerobic, including genera such as *Bacteroides*, *Bifidobacterium*, *Eubacterium*, *Fusobacterium*, and *Peptostreptococcus* (46, 47). In addition, fungi, along with other intestinal microorganisms, are important components of the human gut microbiota that contribute significantly to maintaining gut health and regulating intestinal inflammation (48). Although fungi account for a smaller proportion of the microbiota than bacteria in terms of number, they are significantly larger in size. As a result, they occupy a substantial proportion of the biomass (49). In the human gut, *Ascomycota* and *Basidiomycota* are two dominant phyla of fungi (50). In terms of genera, the community is mostly made up of *Aspergillus*, *Candida*, *Debaryomyces*, *Malassezia*, *Penicillium*, *Pichia*, and *Saccharomyces* (51).

The gut microbiota is dynamic rather than static; its composition is primarily influenced by the mode of delivery at birth and subsequently shaped alongside external environmental factors and behavioral habits, including diet, physical activity, medication use, and psychological stress. Through continuous selection and adaptation in conjunction with the host, the microbiota establishes a stable interdependent state while maintaining dynamic responses to variations in external conditions (52, 53). The harmonious interplay between a healthy intestinal microbial structure, an intact mucosal barrier, and a fully developed immune system is essential for sustaining the dynamic equilibrium of the gut microecosystem. When exposed to adverse external factors, the composition and abundance of microorganisms can change, particularly resulting in dysbiosis characterized by changes in bacterial species, which can significantly impact various aspects of human health (54).

## 3 The potential pro-carcinogenic mechanisms of gut microbiota in CRC

Dysbiosis of the human gut microbiota is closely associated with CRC. Patients with CRC typically exhibit signs of dysbiosis in their gut microbiota, which is characterized by decreased bacterial abundance and diversity in both intestinal mucosa and fecal samples compared to those from healthy individuals (55, 56). Specific pathogenic bacterial species in the gut exhibit a significant increase, such as *Fusobacterium nucleatum*, enterotoxigenic *Bacteroides fragilis*, *Escherichia coli*, *Enterococcus faecalis*, *Streptococcus gallolyticus*, and *Salmonella enterica* (57–59). Generally, the ratio of *Basidiomycota* to *Ascomycota* is often regarded as an index that reflects fungal dysbiosis in an ecosystem (60–62). Compared with healthy subjects, patients with CRC exhibit an increased *Basidiomycota: Ascomycota* ratio and alterations in the gut fungal microbiota. The fungal class *Malasseziomycetes* is enriched, while the classes *Pneumocystidomycetes* and *Saccharomycetes* are depleted in CRC. Six fungal genera are enriched, including *Malassezia*, *Moniliophthora*, *Rhodotorula*, *Acremonium*, *Thielaviopsis*, and *Pisolithus* (63). In a meta-analysis involving data from eight cohorts, researchers identified six fungal species that were consistently enriched in fecal samples of CRC, including *Aspergillus rambellii*, *Cordyceps* sp.*RAO-2017*, *Erysiphe pulchra*, *Moniliophthora perniciosa*, *Sphaerulina musiva*, and *Phytophthora capsici*. Incidentally, the enteric fungi and bacteria demonstrated transkingdom interactions in the progression of CRC, with *Aspergillus rambellii* exhibiting a strong association with the CRC-enriched bacterium *Fusobacterium nucleatum*. *Aspergillus rambellii* was found to promote CRC cell proliferation *in vitro* and to enhance tumor growth in xenograft mouse models (64). Moreover, *Candida albicans* is one of the most studied fungi in relation to human health, with evidence indicating a significantly increasing trend in the feces of patients with CRC (65). To date, a growing body of research has demonstrated that these microorganisms can promote the initiation and progression of CRC through various mechanisms, including the following.

### 3.1 Chronic inflammation and oxidative stress

Chronic inflammation can be initiated by biological, chemical, and physical factors and is associated with an increased risk of various human malignancies (66). It is estimated that approximately 20% of tumors are preceded by chronic inflammation (67). During carcinogenesis, inflammation can release cytokines, generate free radicals, damage DNA, promote cell proliferation, and induce angiogenesis, ultimately facilitating tumor progression (68).

Inflammation serves as a critical environmental trigger influencing the composition of the microbiota, and chronic inflammation is recognized as a significant marker of colorectal carcinogenesis (69). Dysbiosis of the gut microbiota can lead to dysfunction of the intestinal mucosal barrier, allowing harmful substances and toxins to infiltrate intestinal tissues, thereby triggering inflammatory responses that promote tumorigenesis and further create a favorable environment for tumor growth and metastasis (28).

Chronic inflammation stimulates tumor growth and development through key cytokines, such as nuclear factor kappa-B (NF-κB) and signal transducer and activator of transcription 3 (STAT3) (70). These factors enhance the production of additional pro-tumor cytokines and chemokines, leading to leukocyte recruitment, inducing cell proliferation, angiogenesis, lymphangiogenesis, and tumor cell invasion (71). Inflammatory cytokines and chemokines can attract immature myeloid cells or pro-inflammatory helper T cells, mediating angiogenic factors and tissue remodeling enzymes, and suppressing anti-tumor T cell responses, thereby influencing tumor progression (72). Moreover, chronic inflammation can induce tissue damage and oxidative stress (OS), leading to progressive accumulation of DNA damage in epithelial cells, ultimately resulting in the malignant transformation of intestinal epithelial cells (68).

Dysbiosis of the gut microbiota can trigger inflammatory responses that promote carcinogenic pathways, with key cytokines primarily including IL-1β, IL-6, IL-17, IL-22, IL-23, and TNF-α (73–78). When gut microbiota dysbiosis occurs, the proliferation of pathogenic bacteria increases endotoxins and other Gram-negative bacterial components. This activates the systemic inflammatory cascades, resulting in elevated secretion of IL-6, TNF-α, and other pro-inflammatory cytokines, thereby triggering an inflammatory response (68). IL-6 stimulates the STAT3 signaling pathway, inducing the expression of anti-apoptotic factors Bcl-xL and Bcl-2, resulting in the aberrant accumulation of T cells in the intestinal mucosa, further exacerbating the inflammatory response and accelerating the progression of adenomas to CRC (79). Moreover, gut microbiota may activate Toll-like receptors (TLRs), leading to increased expression of IL-1β and TNF-α while promoting the production of COX-2. These cytokines mediate the synthesis of prostaglandin E2, enhancing the inflammatory response and promoting carcinogenesis (68). Myeloid differentiation factor 88 (MyD88) is a critical adaptor molecule in the TLRs signaling pathway. MyD88 mediates the production of inflammatory cytokines such as IL-23 and IL-6, inducing differentiation of Th17 immune cells, upregulating IL-17 and IL-22, and ultimately promoting tumor cell proliferation by activating signaling pathways, including NF-κB and STAT3 (80–82). Additionally, **Table 1** provides details about specific pathogenic bacteria in the gut that mediate the production of pro-inflammatory cytokines, subsequently triggering inflammation (83–94).

**Table 1:** Specific pathogenic bacteria in the gut triggering inflammation through pro-inflammatory cytokines.

Author	Year	Experimental subjects	Pathogenic bacteria	Pro-inflammatory cytokines
Duizer, C., et al. (83)	2025	HT-29 cells	*Fusobacterium nucleatum*	CXCL8 (IL-8)
Yu, Y., et al. (84)	2025	HT-29 cells	*Fusobacterium nucleatum*	IL-8
Bostanghadiri, N., et al. (85)	2023	human	*Fusobacterium nucleatum*	IL-6 and TNF-α
Martin-Gallausiaux, C., et al. (86)	2024	HCT116 and HT-29 cells	*Fusobacterium nucleatum*	IL-8
Zhang, L., et al. (87)	2023	HCT116 and HT-29 cells	*Enterococcus faecalis*	IL-8
Cavallucci, V., et al. (88)	2022	CSC-P cells	*Fusobacterium nucleatum*	CXCL-1 and IL-8
Yin, H., et al. (89)	2022	mice	*Fusobacterium nucleatum*	IFN-γ, TNF-α, IL-6, IL-12, IL-17A, CXCL1, IL-9, MCP-1 and Eotaxin
Purcell, R. V., et al. (90)	2022	HCT116 and HT-29 cells	*Bacteroides fragilis*	IL-8
Cuellar-Gómez, H., et al. (91)	2022	human	*Fusobacterium nucleatum*	IL-23 and IL-17
Kim, Y. J., et al. (92)	2021	mice	*Fusobacterium nucleatum*	IL-1β and TNF-α
Xie, X., et al. (93)	2021	SW620 and HT-29 cells	*Bacteroides fragilis*	CCL3
Ma, C. T., et al. (94)	2018	NCM460 cells	*Fusobacterium nucleatum*	IL-6 and IL-1β

**Figure 1:**
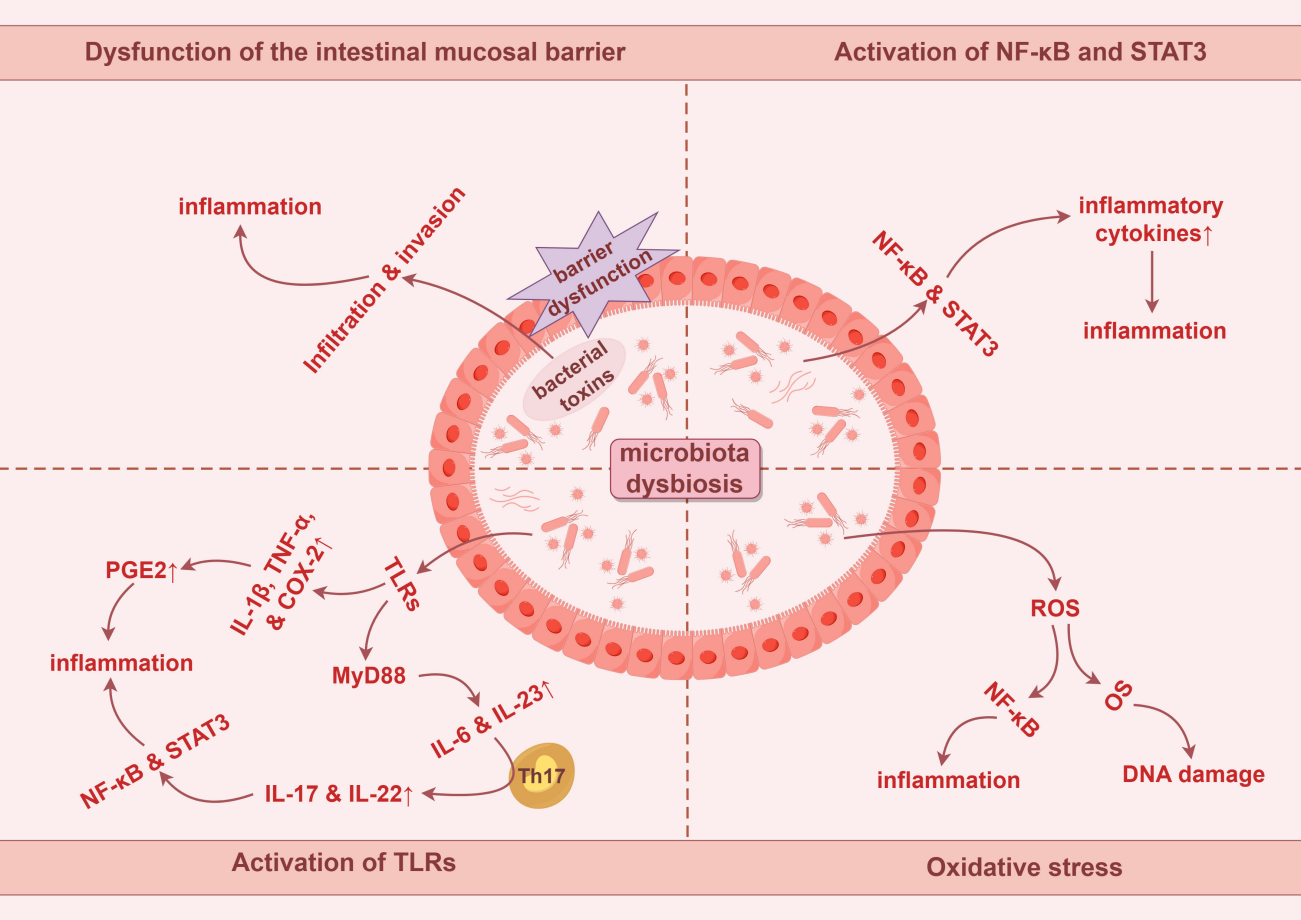
Potential tumorigenic mechanisms of intestinal microbiota dysbiosis in colorectal cancer involve chronic inflammation and oxidative stress. PGE2, prostaglandin E2. By Figdraw.

OS is widely acknowledged to refer to the imbalance between reactive oxygen species (ROS) and reactive nitrogen species (RNS) within cells, typically manifested by the production of ROS and RNS exceeding the clearance capacity of the antioxidant system, thus contributing to cellular damage and the development of various diseases. The interaction between intestinal epithelial cells and specific pathogenic bacteria can stimulate the production of ROS. For instance, *Enterococcus faecalis* can induce macrophages to generate ROS, leading to superoxide formation and DNA damage in epithelial cells. This process, mediated by the NF-κB signaling pathway, orchestrates inflammation and interferes with the progression of CRC (95). The potential inflammatory mechanisms of pathogenic bacteria in CRC are illustrated in **Figure 1**.

In addition, intestinal fungi may also be involved in the occurrence and development of CRC through inflammatory pathways. Researchers have observed that high concentrations of *Candida* in gastrointestinal tumors can promote its colonization through inflammatory responses, while *Candida* itself can sustain an inflammatory environment linked to the activation of inflammatory signaling pathways mediated by the pro-inflammatory factor IL-1 and neutropenia (96). *Candida albicans* is one of the most common opportunistic fungal pathogens (97). It may facilitate cancer development through multiple mechanisms, including the disruption of the mucosal epithelium, the elicitation of chronic inflammation, and the induction of Th17 immune responses (98). Intestinal epithelial cells express dectin-1, a molecule associated with the antifungal responses of myeloid cells and intestinal inflammation. Intestinal epithelial cells may recognize the activation of the Wnt pathway by *Candida albicans* through dectin-1 to facilitate the development of CRC (99). Additionally, *Candida tropicalis* can promote CRC by activating the NLRP3 inflammasome through glycogen metabolism-dependent glycolysis and the JAK/STAT1 signaling pathways (100). *Candida* is suspected to participate in the progression of CRC by inducing inflammation.

### 3.2 Toxins derived from gut microbiota

In the context of gut dysbiosis, various pathogenic bacteria in the gut can secrete a range of toxins. These toxins not only induce DNA damage in host cells, leading to mutations or deletions in oncogenes and tumor suppressor genes, but also modulate host signaling pathways, thereby promoting the onset and progression of CRC directly or indirectly (57). *Fusobacterium nucleatum* produces multiple virulence factors, among which *Fusobacterium adhesion* protein A (FadA) binds to E-cadherin on intestinal epithelial cells, activating the Wnt/β-catenin signaling pathway. This activation results in the nuclear translocation of β-catenin, leading to the overexpression of inflammatory and oncogenic genes, such as C-MYC and CCND1, driving malignant cell proliferation and tumor formation (57, 101). Additionally, *Fusobacterium* autotransporter protein 2 (Fap2), another adhesin produced by *Fusobacterium nucleatum*, binds to the inhibitory immune receptor TIGIT (T cell immunoreceptor with Ig and ITIM domains), which diminishes the activity of natural killer cells (NK cells) and T cells. Such a reduction in immune activity subsequently inhibits immune responses, promotes immune cell apoptosis, and facilitates the evasion of cancer cells from immune surveillance (102).

BFT, a 21 kDa zinc-dependent metalloproteinase toxin produced by enterotoxigenic *Bacteroides fragilis*, interacts with receptors on colonic epithelial cells, thereby compromising intestinal epithelial barrier function. It activates the Wnt/β-catenin and NF-κB signaling pathways, resulting in increased cell proliferation, DNA damage, and the release of inflammatory mediators, especially IL-17 (103). BFT also influences immune cells by activating the TLRs signaling pathway, which upregulates IL-6 and TNF-α, further leading to the activation of STAT3 and NF-κB, thereby inhibiting anti-tumor immunity and promoting tumorigenesis (68).

**Figure 2:**
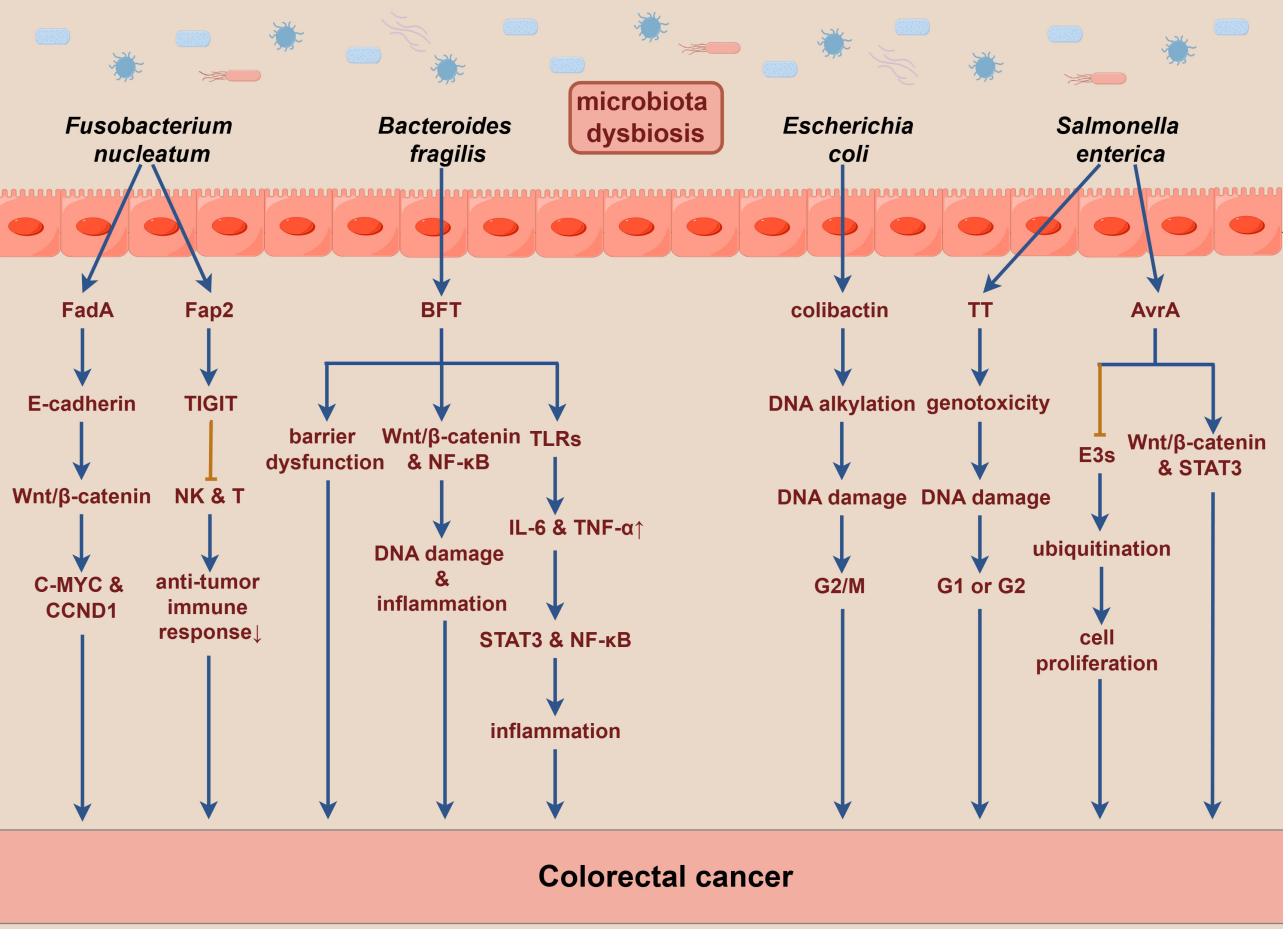
Toxins produced by gut microbiota may significantly contribute to the pathogenesis of colorectal cancer. E3s, E3 ubiquitin ligases. By Figdraw.

Moreover, *Escherichia coli* strains containing the polyketide synthetase genes produce specific toxins such as colibactin that induce DNA alkylation and DNA adduct formation, leading to genotoxicity. Such alterations lead to DNA double-strand breaks and cell cycle arrest at the G2/M phase, resulting in chromosomal abnormalities. Subsequently, incomplete DNA repair may promote tumorigenesis (104, 105). *Enterococcus faecalis* produces metalloproteinases, such as gelatinase, which can directly compromise the intestinal epithelial barrier and trigger inflammation (106). The typhoid toxin (TT) secreted by *Salmonella enterica* shares similarities with the toxins of *Escherichia coli*, exhibiting genotoxicity that induces DNA damage responses, resulting in cell cycle arrest at the G1 or G2 phase, thereby triggering cellular senescence or apoptosis (107). Furthermore, *Salmonella* Anti-Virulence Agent A (AvrA), an effector secreted by *Salmonella enterica*, inhibits the activity of E3 ubiquitin ligases. This inhibition interferes with protein ubiquitination and modulates the host immune response, which leads to reduced cellular apoptosis and promotes intestinal cell proliferation, ultimately contributing to increased tumorigenesis. Additionally, AvrA activates the Wnt/β-catenin and STAT3 signaling pathways, further facilitating colorectal carcinogenesis (108). The underlying mechanisms discussed above are described in detail in **Figure 2**.

### 3.3 Metabolites derived from gut microbiota

The gut microbiota plays a crucial role in host’s digestion and nutrient absorption by anaerobically fermenting indigestible food substrates into various metabolites. These metabolites interact with the epithelial cells in the intestinal mucosa, modulating immune responses and potentially contributing to the onset and progression of intestinal diseases (109). Dysbiosis of the gut microbiota can lead to alterations in microbiota-derived metabolite levels, significantly influencing the development of CRC (110). Certain metabolites are recognized for their distinct carcinogenic properties, primarily as nitrogen compounds, hydrogen sulfide, and secondary bile acids (SBAs).

Research has shown that fermentation of aromatic amino acids by gut bacteria produces a range of toxic metabolites, such as ammonia, amines, and sulfides, which may contribute to the onset and progression of CRC by inducing chronic inflammation and causing DNA damage in tissue (111). Specific nitrogen-containing compounds, particularly nitrites, have been shown to elevate cancer risk by inducing DNA alkylation (112). Ammonia is also considered a potential carcinogen at low concentrations, with existing animal model studies demonstrating its association with intestinal mucosal damage and CRC development (113). Polyamines exhibit toxicity at elevated levels and are associated with OS and various diseases, including cancer. OS induced by polyamine catabolism is a key mechanism underlying their toxicity (114).

Specific pathogenic bacteria such as *Shigella flexneri*, *Streptococcus pneumoniae*, *Helicobacter pylori*, and *Salmonella enterica serotype Typhi* utilize polyamines to enhance virulence (115). Ornithine decarboxylase is a critical enzyme that is involved in the polyamine biosynthetic pathway, with expression significantly elevated in CRC tumor tissues compared to adjacent normal mucosa, suggesting that increased polyamines may contribute to CRC pathogenesis (116). Furthermore, polyamines are integral in carcinogenic signal transduction; elevated levels of spermidine and spermine can lead to the upregulation of β-catenin expression, promoting tumor cell proliferation, invasion, and metastasis. This mechanism has been corroborated by studies in various cancers, including CRC (117).

Sulfate-reducing bacteria, such as *Desulfovibrio*, *Desulfobacter*, and *Clostridium*, colonize the human gut and produce endogenous hydrogen sulfide through the metabolism of both inorganic and organic sulfur compounds (118). Hydrogen sulfide can generate polysulfide via a series of mitochondrial enzyme-catalyzed reactions, which not only inhibits butyrate oxidation and compromises colonic mucosal barrier integrity, but also induces DNA damage mediated by ROS, thereby promoting tumor progression (119). Existing studies suggest that sulfides are involved in post-translational modifications, activating the RAS-RAF-MEK-ERK signaling pathway through the thiolation of MEK1, which consequently regulates DNA damage repair mechanisms and influences tumor growth. Additionally, hyper-sulfation of NF-κB may enhance the expression of metastasis-related genes, activate the NF-κB/IL-1 signaling pathway, and promote tumor progression and metastasis through vascular endothelial growth factor (VEGF) activation (120).

**Figure 3:**
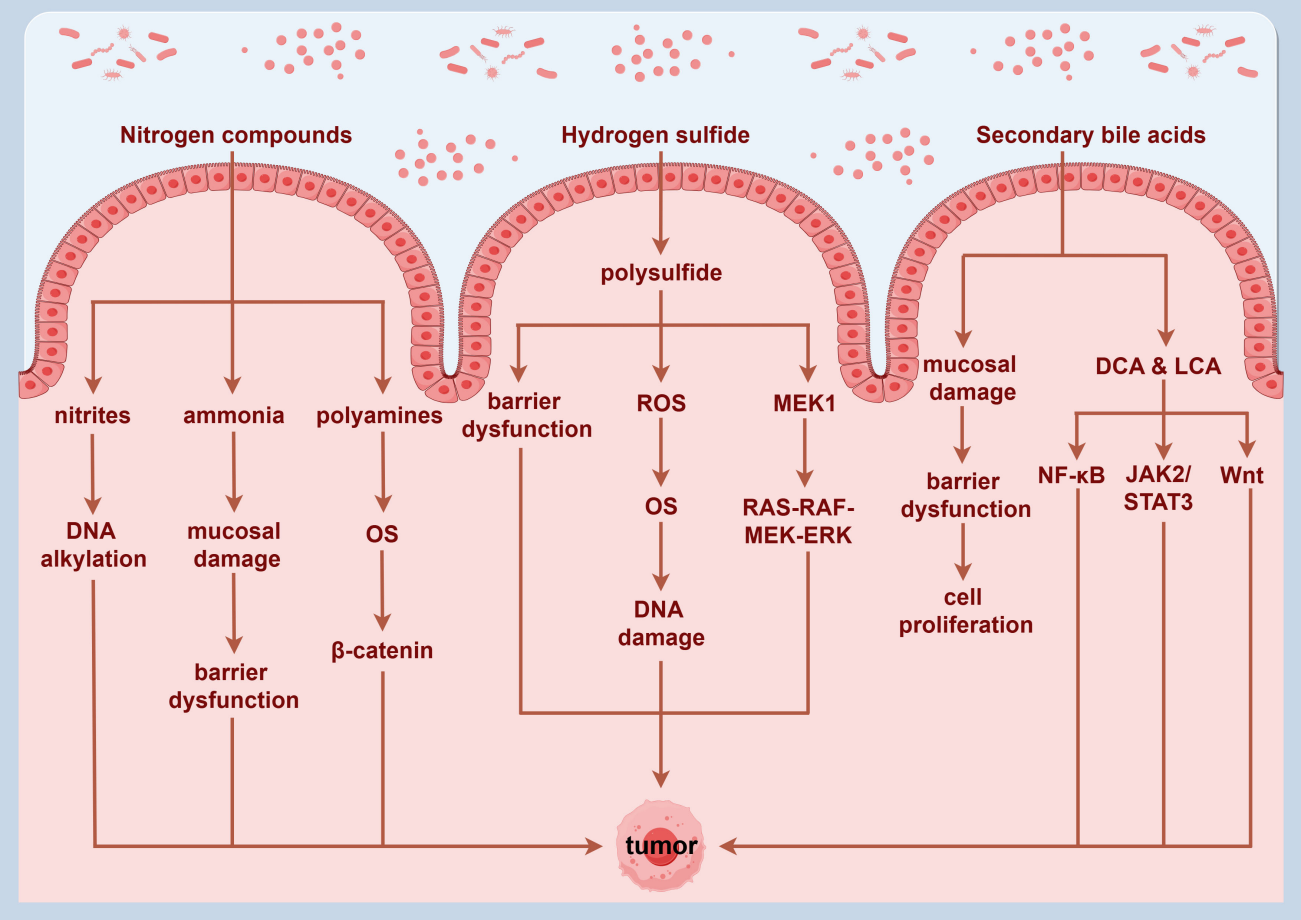
Potential mechanisms through which metabolites derived from gut microbiota may promote the development of colorectal cancer. By Figdraw.

Moreover, the gut microbiota plays a pivotal role in the metabolism of bile acids, converting primary bile acids (PBAs) derived from the liver into SBAs, mainly consisting of deoxycholic acid (DCA) and lithocholic acid (LCA). Alterations in bile acid metabolism and composition are closely associated with CRC (121). Bile acids exhibit a bidirectional regulatory effect on human physiology. At physiological concentrations, SBAs can exert immunomodulatory and anti-inflammatory effects, contributing to the suppression of inflammatory bowel diseases. However, elevated concentrations of SBAs may compromise intestinal epithelial integrity, inducing excessive proliferation of undifferentiated cells, and increasing the risk of precancerous conditions (122). Recent studies have indicated that the malignant transformation of colorectal adenomas may be closely linked to the interaction between bile acids and gut microbiota. DCA and LCA may promote CRC development by modulating the NF-κB and JAK2/STAT3 signaling pathways (123). Additionally, DCA can activate the Wnt signaling pathway, triggering inflammatory responses and promoting cellular proliferation, both of which are critically significant in CRC pathogenesis and progression. Furthermore, DCA influences tumor growth by facilitating the release of β-catenin, which accumulates in the cytoplasm before translocating to the nucleus, where it activates transcription factors, including T-cell factor and lymphoid enhancer factor, thereby exerting oncogenic effects (124). Metabolites derived from the gut microbiota that are linked to CRC are shown in **Figure 3**.

### 3.4 Epigenetic dysregulation

CRC is characterized by distinct molecular alterations that can be broadly classified into two primary categories: the first involves mutations that enhance the activity of oncogenes, while the second pertains to mutations leading to the loss of function of tumor suppressor genes. The pathogenesis and progression of CRC are driven by a variety of genetic alterations, notably including chromosomal instability (CIN), microsatellite instability (MSI), the CpG island methylator phenotype (CIMP), and various epigenetic modifications. The mechanisms underlying these epigenetic alterations encompass DNA methylation, histone modifications, and regulation by microRNAs (123).

Approximately 65%-70% of sporadic CRC cases exhibit CIN, characterized by mutations in oncogenes (such as KRAS and BRAF) and tumor suppressor genes (like APC and TP53), as well as chromosomal aberrations, including the deletion of chromosome 18q. In contrast, only around 15% of sporadic CRC cases display MSI, primarily because of defects in genes encoding DNA mismatch repair proteins, including MLH1, MSH2, MSH6, and PMS2. These defects result in a markedly increased mutation rate within colonic mucosal cells (125). CIMP is closely associated with hypermethylation of the promoter regions of tumor suppressor genes, which manifests as aberrant methylation across multiple CpG sites within the tumor genome. Initially identified in CRC patients, CIMP is considered a distinctive mechanism contributing to the pathogenesis and progression of this malignancy. A hallmark of CIMP is the significantly elevated frequency of methylation in promoter regions, which leads to the silencing of tumor suppressor genes (123, 126).

DNA methylation frequently occurs in the promoter regions of tumor suppressor genes, potentially resulting in their silencing and facilitating cancer progression. Emerging research suggests that gut microbiota play a pivotal role in this process by influencing host metabolism, immune responses, and gene expression, which may indirectly affect DNA methylation status and that are thus associated with the onset and progression of cancer (127). For instance, *Fusobacterium nucleatum* has been shown to inhibit T cell-mediated immune responses and modulate genetic mutations in key oncogenes and tumor suppressor genes, including BRAF, KRAS, TP53, CHD7, and CHD8. These mutations may induce cancer-associated methylation phenotypes such as CIMP and increase MSI, ultimately contributing to CRC progression (128). Furthermore, recent studies have reported that in CIMP-positive CRC, *Fusobacterium nucleatum* can induce hypermethylation in the promoter regions of tumor suppressor genes such as MTSS1, PKD1, PTPRT, MLH1, CDKN2A, and EYA4, leading to their silencing. This process is thought to be closely linked to the release of hydrogen sulfide, inflammatory cytokines, ROS, and the recruitment of DNA methyltransferases (123).

*Hungatella hathewayi*, a Gram-negative, anaerobic, spiral-shaped bacterium, has also been implicated in the hypermethylation of genes such as SOX11, THBD, SFRP2, GATA5, ESR1, EYA4, CDX2, and APC. The recruitment of DNA methyltransferases may represent a mechanism through which this microorganism induces methylation (58). Additionally, *Streptococcus gallolyticus* has been associated with the hypermethylation of critical tumor suppressor genes, specifically MLH1 and APC (129). Furthermore, *Parvimonas micra* is an oral pathobiont implicated in CRC. It may induce chronic inflammatory responses and dysregulation of host immune responses. These effects can potentially lead to hypermethylation of essential tumor suppressor genes, including SCIN, HACE1, TSPAN13, FBXO32, IGFBP7, SIX1, and CXXC5 (130). Moreover, certain oral pathogenic bacteria, particularly Gram-negative species, are capable of producing lipopolysaccharides (LPS). Studies have shown that LPS can activate various signaling pathways, including TGF-β and Wnt/β-catenin, by binding to receptors on the surface of host cells. The regulation of these signaling pathways may influence the expression or activity of DNA methylation-related enzymes, thereby indirectly altering DNA methylation patterns (131).

### 3.5 Immune regulatory dysfunction

The gut microbiota play a pivotal role in shaping the immune microenvironment by interacting with the gut immune system, influencing the development and differentiation of immune cells, and modulating signaling pathways that regulate immune functions (132). Most cancers, including CRC, originate from prolonged precancerous lesions, with the host immune system acting as a crucial factor in maintaining the stability of these lesions. Precancerous lesions can evade host immune surveillance through various mechanisms, which directly or indirectly induce immune suppression, thereby facilitating the progression from precancerosis to malignancy (68).

The transition from colorectal adenoma to CRC typically involves three immune stages: elimination, equilibrium, and escape. During the immune elimination phase, precancerous lesions can potentially be eliminated by immune responses, leading to early apoptosis or abnormal differentiation of affected cells. In the immune equilibrium phase, a minority of precancerous lesions evade immune elimination through diverse mechanisms, allowing them to persist. Finally, in the immune escape phase, precancerous cells acquire the ability to evade immune surveillance. This renders them resistant to immune attacks and allows for unrestrained growth, ultimately resulting in malignant tumor development (133).

The intestinal immune environment comprises a diverse array of lymphocytes and myeloid-derived immune cells, which are essential for maintaining local immune balance within the gut and systemic immune homeostasis (134). Dysbiosis of the gut microbiota can disrupt immune homeostasis, potentially leading to the direct or indirect suppression of immune cell functions. This disruption facilitates immune evasion by tumor cells and contributes to the development of CRC (135).

*Fusobacterium nucleatum* can promote CRC progression and metastasis by inhibiting the antitumor immune responses of NK cells and T cells (68). *Fusobacterium nucleatum* may induce the tumor-derived chemokine CXCL1, which recruits myeloid-derived suppressor cells (MDSCs), thereby reducing T cell abundance in the tumor microenvironment and inhibiting antitumor immunity (135, 136). Additionally, succinate produced by *Fusobacterium nucleatum* may inhibit the cyclic GMP-AMP synthase (cGAS) and interferon (IFN) signaling pathway, resulting in decreased levels of the chemokines CCL5 and CXCL10 within the tumor microenvironment. This suppression limits the migration of cytotoxic T lymphocytes to the tumor site and impairs their antitumor function (137).

**Figure 4:**
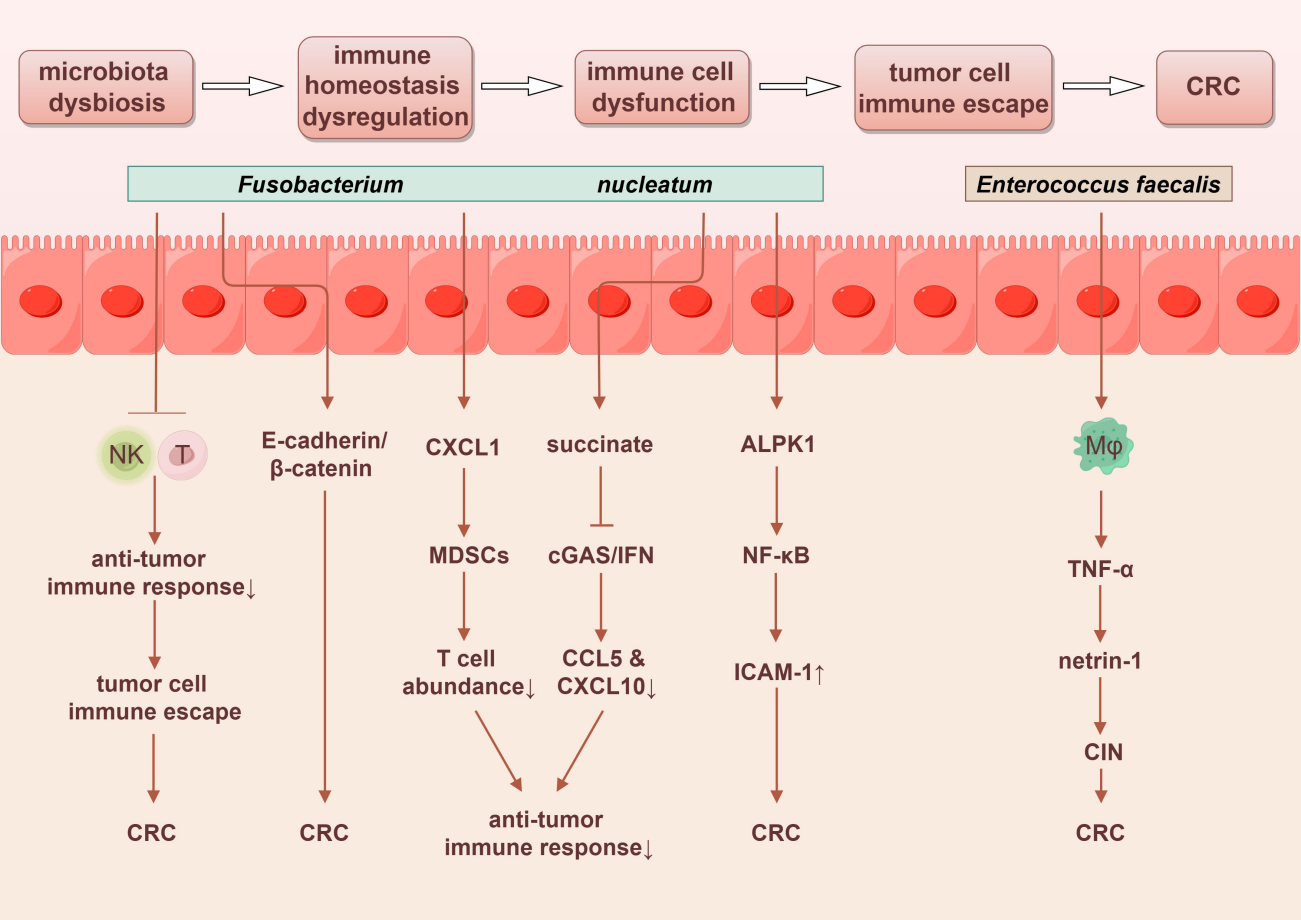
Gut microbiota promotes the progression of colorectal cancer through immune regulatory dysfunction. By Figdraw.

Moreover, intercellular adhesion molecule 1 (ICAM-1), a member of the immunoglobulin superfamily, facilitates the adhesion of tumor cells to endothelial cells, promoting tumor metastasis. *Fusobacterium nucleatum* can activate the NF-κB pathway by interacting with the pattern recognition receptor ALPK1 on host cells, which leads to the upregulation of ICAM-1 expression. This process enhances the adhesion of cancer cells to endothelial cells, thereby mediating CRC metastasis (135). *Enterococcus faecalis* can activate mucosal macrophages, promoting inflammatory responses and inducing a bystander effect. TNF-α secreted by macrophages plays a pivotal role in this process, stimulating the proliferation of colonic epithelial cells through the anti-apoptotic effects of the axon guidance factor netrin-1. When untransformed epithelial cells are exposed to polarized macrophages, this interaction may lead to CIN, thereby increasing the risk of carcinogenesis (68). The possible mechanisms outlined above are visually represented in **Figure 4**.

Furthermore, specific species of fungi also exacerbate the severity of CRC by regulating tumor immunity. For instance, *Candida tropicalis* has been shown to enhance the immunosuppressive capabilities of MDSCs through the Syk-PKM2-HIF-1α-glycolysis signaling pathway, thereby facilitating the progression of CRC (138). Additionally, it elevates the secretion of IL-1β from MDSCs by activating the NLRP3 inflammasome. The subsequent increase in IL-1β levels further amplifies the immunosuppressive functionality of MDSCs, resulting in the inhibition of antitumor immunity and consequently promoting the development of CRC associated with *Candida tropicalis* (139).

## 4 The potential anticancer mechanisms of gut microbiota in CRC

The human gut microbiota encompasses not only pathogenic bacteria but also a diverse array of beneficial symbiotic organisms, including *Lactobacillus*, *Bifidobacterium*, and *Akkermansia* (123). These beneficial bacteria are integral to maintaining intestinal health, facilitating digestion, and enhancing immune function. A relevant study indicated that the abundance of *Lactobacillus acidophilus* in fecal samples from patients with CRC was significantly diminished compared with healthy individuals, highlighting that it may have a protective role in the pathogenesis and progression of CRC (140). *Lactobacillus acidophilus* enhances the expression of intestinal mucosal tight junction proteins, thereby improving intestinal barrier function, resisting pathogenic bacteria, and inhibiting inflammatory response (141–143). Additionally, *Lactococcus lactis* in the gut may play a role in preventing microbiota dysbiosis, stimulating host immune defense mechanisms, and enhancing the expression of IL-18. This cascade of effects contributes to its anti-inflammatory and anti-tumor properties while inhibiting the proliferation of CRC cells (144).

Interferon-inducible protein 10 (IP-10), also known as CXCL10, is a chemokine induced by IFN-γ that plays a pivotal role in immune regulation and inflammatory responses. *Lactobacillus* species have been demonstrated to secrete lactosepins to degrade this chemokine, thereby attenuating the inflammatory response (128). Both *Lactobacillus* and *Bifidobacterium* can metabolize linoleic acid, alleviating inflammation through the inhibition of macrophage activation and the modulation of peroxisome proliferator-activated receptor-gamma (PPAR-γ) expression (128). Furthermore, they promote the renewal of intestinal epithelial cells, reduce the accumulation of Th17 cells, modulate the expression of major histocompatibility complex class II (MHC II) on dendritic cells, enhance the recruitment and activity of NK cells and cytotoxic T cells, and mitigate DNA damage induced by OS. Collectively, these actions contribute to the inhibition of tumor occurrence and progression (57).

The non-enterotoxigenic *Bacteroides fragilis* may enhance intestinal immunity through its immunogenic capsular components, thereby inhibiting chronic colitis and CRC, contrasting with the carcinogenic properties of the enterotoxigenic *Bacteroides fragilis* (145). Moreover, mucin 2 (MUC2), a glycoprotein-rich mucin predominantly secreted by goblet cells, provides adhesion sites for antimicrobial proteins and commensal microbiota within the intestinal lumen. This mucin plays a critical role in defending against the invasion of pathogenic bacteria and harmful substances. *Akkermansia muciniphila* has been shown to exert a protective effect by strengthening the integrity of the intestinal mucosal layer and restoring normal levels of MUC2, thereby inhibiting the development and progression of CRC (146).

Not all metabolites produced by the gut microbiota promote carcinogenesis; rather, certain metabolites exhibit anti-inflammatory and anticancer properties, which help to promote intestinal health and inhibit tumorigenesis. SCFAs, metabolic byproducts generated through the fermentation of dietary fiber by gut anaerobic microorganisms, primarily include acetate, propionate, and butyrate. These SCFAs serve as the primary energy source for colonic epithelial cells and play a crucial regulatory role in local immune responses (117). Key bacterial genera involved in the production of SCFAs include *Bifidobacterium*, *Faecalibacterium*, and *Ruminococcus*, and these are vital for maintaining gut health (123).

SCFAs are instrumental in preserving intestinal mucosal barrier function by promoting mucus production and the expression of the tight junction proteins. Furthermore, they enhance microbial diversity, thereby supporting the overall health of the gut microbiota (147). SCFAs exert anti-inflammatory effects by binding to G protein-coupled receptor 43 (GPR43) on immune cells, such as macrophages, thereby contributing to the maintenance of homeostasis between intestinal immunity and inflammatory responses (117). A substantial body of evidence has demonstrated the anticancer properties of SCFAs, particularly butyrate (128).

Butyrate enhances the expression of the tight junction proteins in intestinal epithelial cells, thereby improving intestinal barrier function. It exerts anti-inflammatory and antitumor effects through mechanisms involving the regulation of cellular metabolism, immune responses, and epigenetic modulation (128, 148). Notably, *Clostridium butyricum*, a well-known butyrate-producing probiotic, effectively modulates the composition of gut microbiota, decreases pathogenic bacteria in the intestine, and reduces the levels of pro-inflammatory cytokines such as IL-1β, IL-6, and TNF-α (149). *Clostridium butyricum* significantly inhibits signaling pathways, for example, MYD88, NF-κB, and Wnt/β-catenin, consequently preventing the development of colorectal inflammation-associated CRC (150, 151).

Butyrate interacts with G protein-coupled receptor 109A (GPR109A), leading to the downregulation of anti-apoptotic proteins such as Bcl-2 and Bcl-xL, and cyclin D1 in CRC cells, while simultaneously upregulating apoptotic receptor signaling pathways, which enhances apoptosis in CRC cells. Additionally, butyrate mitigates tumor-associated inflammatory responses by inhibiting the NF-κB signaling pathway (123). It may also inhibit the AKT signaling pathway through GPR109A activation, thereby reducing the aerobic glycolysis necessary for CRC cell survival and ultimately impeding the proliferation of cancer cells (152).

Moreover, butyrate has been shown to inhibit several oncogenic signaling pathways, such as mitogen-activated protein kinase 1 (MAPK1) and small mothers against decapentaplegic homolog 3 (SMAD3), that are closely associated with the pathogenesis and progression of CRC by regulating cellular proliferation and apoptosis (153). Furthermore, butyrate induces the expansion of regulatory T cells (Treg cells), modulating local immune responses and inhibiting colonic inflammation, thereby reducing the risk of colonic carcinogenesis (154). The anticancer properties of butyrate may also be demonstrated by its ability to regulate the methylation status of the promoters of the tumor suppressor gene ABCA1 and the oncogene EGR3, both implicated in cancer development. Notably, butyrate’s regulatory effect exhibits a dose-dependent relationship (155).

**Figure 5:**
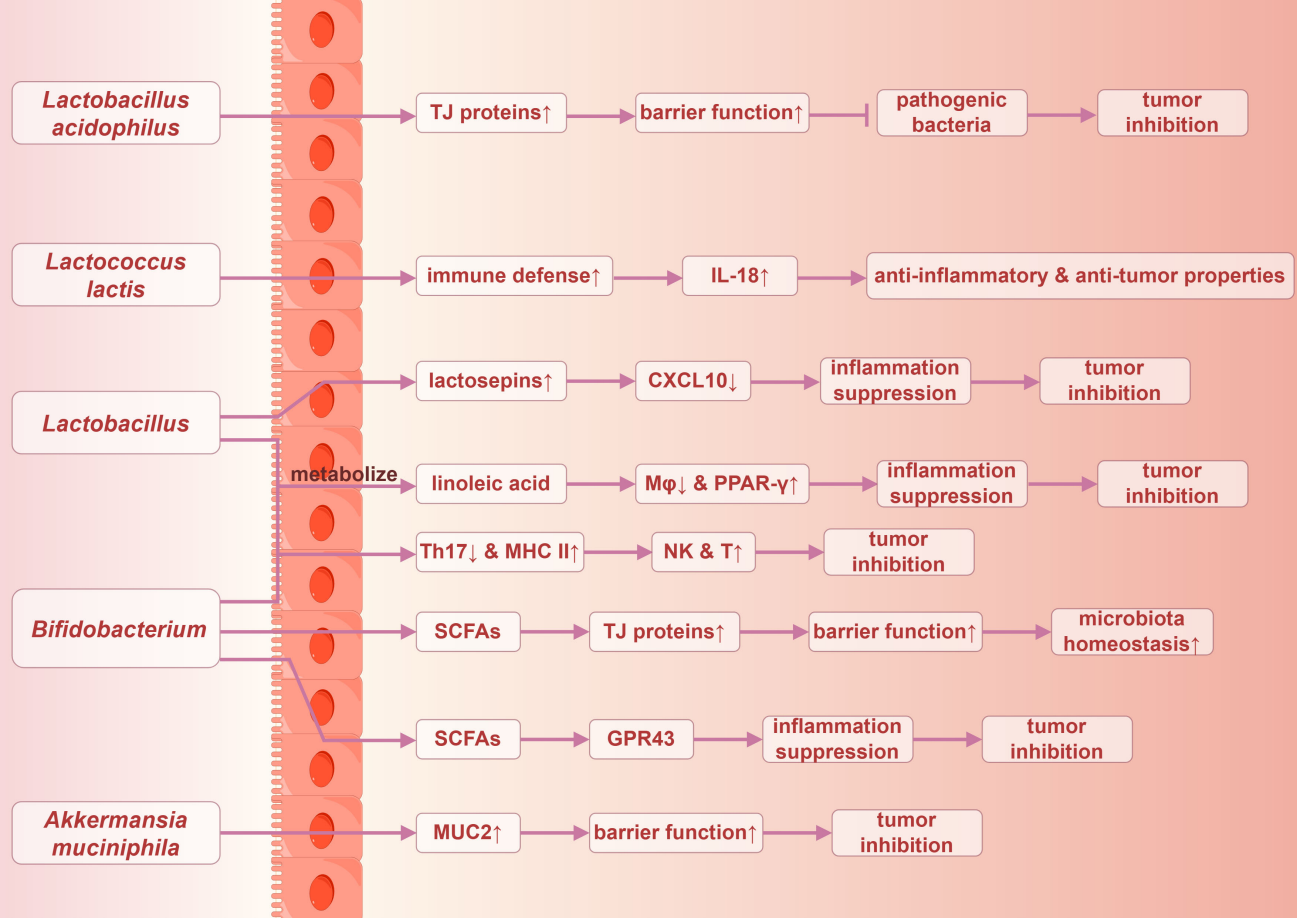
Possible anticancer mechanisms of gut microbiota in colorectal Cancer. TJ proteins, tight junction proteins. By Figdraw.

Additionally, B vitamins can be obtained through dietary intake and by gut microbiota metabolism, potentially exerting a preventive effect against the risk of CRC. A relevant study indicated that vitamin B3 interacted with G protein-coupled receptors (GPRs) and prostaglandin receptors to inhibit inflammatory and carcinogenic processes. By activating the GPR109A signaling pathway, vitamin B3 enhanced the anti-inflammatory effects in colonic macrophages and dendritic cells, subsequently activating Treg cells, thereby suppressing tumor initiation and progression (123). The potential anticancer mechanisms of gut microbiota are succinctly summarized in **Figure 5**.

## 5 Gut microbiota modulation: a promising strategy for CRC prevention and treatment

To date, a considerable amount of evidence has indicated that the modulation of gut microbiota plays a crucial role in effectively preventing and treating CRC (156–159). A variety of strategies can be employed to achieve this modulation, including dietary interventions that promote a diverse and balanced microbial ecosystem, probiotic supplementation that introduces beneficial live microorganisms, the application of natural plant extracts known for their bioactive compounds that exhibit anti-cancer properties, and fecal microbiota transplantation. These approaches can improve gut health, leading to a reduction in CRC incidence and better outcomes for patients following cancer treatment.

### 5.1 Prevention of CRC through modulation of the gut microbiota

Dietary patterns play a critical role in shaping the gut microecosystem and are closely associated with gastrointestinal diseases. A pertinent study demonstrated that a high-fiber diet can significantly reduce susceptibility to CRC (160). Specifically, adherence to a whole grain diet had been associated with a decreased risk of CRC; an increase of 90 grams of whole grains consumed daily correlated with a 17% reduction in CRC incidence (161). Dietary fiber enhances the abundance of bacteria responsible for the production of SCFAs, alters microbial community composition, and increases levels of protective metabolites. These effects collectively improve intestinal mucosal barrier function and inhibit specific carcinogenic pathways, thereby attenuating the incidence and progression of CRC (162). A diet rich in whole grains and dietary fiber was also associated with a lower risk of *Fusobacterium nucleatum*-associated CRC, suggesting a potential anti-cancer effect through the inhibition of the abundance of pathogenic bacteria (163).

The mechanisms by which dietary fiber contributes to CRC prevention are likely multifaceted. Firstly, increased fiber intake enhances intestinal motility, thereby shortening transit time and reducing the duration of contact between carcinogens and the colonic mucosa. Secondly, increased fecal bulk dilutes the concentration of carcinogens within the gut, further mitigating their potential harm. Additionally, the fermentation of dietary fiber boosts the production of SCFAs, particularly butyrate, while simultaneously inhibiting the synthesis of SBAs. Moreover, dietary fiber may lower colonic pH, suppressing the proliferation of pathogenic bacteria while promoting the growth of beneficial microbiota, ultimately reducing CRC risk (116).

Furthermore, whole milk and other dairy products are rich in calcium and various micronutrients. A relevant study identified a significant inverse relationship between calcium intake and the risk of colorectal adenomas, suggesting that adequate calcium consumption may contribute to a reduction in the incidence of colorectal precursors (164). A low-calcium diet may lead to dysbiosis of the gut microbiota, whereas high calcium supplementation can restore the ecological balance of the gut microbiome and increase the abundance of beneficial strains such as *Lactobacillus reuteri*, *Lactobacillus plantarum*, *Lactobacillus bulgaricus*, and *Streptococcus thermophilus*, thereby enhancing potential anti-tumor activity (122). Calcium may bind to SBAs in the intestine, thereby reducing the carcinogenic exposure of epithelial cells to these substances, inhibiting cell proliferation and inducing apoptosis, which leads to tumor suppression (123).

### 5.2 Treatment of CRC through modulation of the gut microbiota

Existing evidence supports that dietary interventions focused on fiber intake may provide substantial benefits and contribute to prolonged survival in patients with CRC (165). Moreover, higher calcium intake has been found to be positively correlated with improved survival rates, suggesting that increased calcium consumption is associated with better overall survival and disease-free survival rates (166). Furthermore, probiotic supplementation, aiming to enhance the abundance of beneficial strains and improve gut microbiota composition, may play a significant role in inhibiting the development of CRC.

Probiotic consumption has been linked to the regulation of gut microbiota, alleviation of chronic inflammation, enhancement of anticancer metabolites, and modulation of immunity, thereby potentially exerting anti-tumor effects (167, 168). As a promising adjunctive therapy, probiotics offer multiple benefits, including a decreased incidence of anastomotic leakage, shortened gastrointestinal recovery time, and reduced chemotherapy-related side effects. These advantages ultimately lower the risk of complications and enhance surgical success rates (169, 170). Moreover, probiotics significantly reduce the incidence of surgical site infections and shortens the duration of hospital stays. Interventions utilizing multiple strains of probiotics are more effective in decreasing postoperative infections compared to those employing a single strain (171). During the perioperative period, the usage of probiotics maintains intestinal mucosal integrity, reduces bacterial translocation, and promotes a more favorable balance between beneficial and pathogenic microorganisms (172).

Furthermore, certain flavonoids and bioactive polyphenols derived from natural plants, such as quercetin, luteolin, anthocyanins, apigenin, curcumin, and resveratrol, exhibit significant anti-inflammatory, antioxidant, antibacterial, and immunomodulatory properties. These natural extracts can modulate gut microbiota composition, promoting the growth of beneficial bacteria and maintaining intestinal health. Their anticancer mechanisms include the reduction of cell proliferation, induction of apoptosis, inhibition of angiogenesis, and delay of tumor metastasis. Additionally, these agents may serve as preventive measures against CRC and enhance chemotherapeutic efficacy or mitigate side effects (116, 123, 173, 174).

In addition, fecal microbiota transplantation involves transferring intestinal microbiota from a healthy donor to the gastrointestinal tract of a recipient, aiming to restore microbial diversity and reconstruct microbial structure for therapeutic outcomes in gastrointestinal diseases (75). Fecal microbiota transplantation is a critical intervention for reestablishing intestinal microbiota homeostasis and has demonstrated significant therapeutic efficacy. Notably, a related study indicated that the combined use of fecal microbiota transplantation with programmed cell death protein 1 (PD-1) inhibitors may yield synergistic effects, enhancing the efficacy of anticancer therapies and improving survival rates among CRC patients (175). Moreover, Fecal microbiota transplantation has been shown to inhibit the development of CRC by reversing gut microbial dysbiosis, alleviating excessive intestinal inflammation, and strengthening anti-cancer immune responses (176). It may become a cornerstone of CRC treatment, emphasizing the urgent need for ongoing research and clinical validation.

## 6 Conclusions and future directions

In summary, the gut microbiota is intricately linked to CRC, significantly influencing its initiation and progression. The role of the gut microbiota in CRC is multifaceted, as it can both promote and inhibit tumor development. Tumor promotion may occur through various mechanisms, including chronic inflammation, the production of toxins and metabolites by gut microbiota, epigenetic dysregulation, and immune regulatory dysfunction. Conversely, tumor inhibition may result from the activity of beneficial microorganisms that produce protective substances, such as SCFAs, and enhance intestinal barrier integrity. Despite substantial advancements in current research, the complex underlying mechanisms remain incompletely elucidated, highlighting the need for further investigation to unravel these intricacies.

Moreover, strategies to modulate gut microbiota, such as probiotic supplementation and natural plant extracts, hold significant promise as novel therapeutic approaches for CRC. However, translating these strategies into established clinical treatments requires extensive foundational research and rigorous clinical trials. Such efforts are crucial for the effective implementation of these anticancer therapies. Consequently, this area is poised to be a focal point for ongoing and future research. These efforts have the potential to drive innovation and significantly improve CRC treatment strategies.
